# Supplementary material for: Functional Characterization and Synthetic Application of Is2-SDR, a Novel Thermostable and Promiscuous Ketoreductase from a Hot Spring Metagenome
Source: Int J Mol Sci. 2022 Oct 12;23(20):12153. doi: 10.3390/ijms232012153 (PMC9603792; doi:10.3390/ijms232012153)

Supporting Information

Functional Characterization and Synthetic Application of Is2-SDR, a Novel Thermostable and Promiscuous Ketoreductase from a Hot Spring Metagenome

Erica Elisa Ferrandi [1], Ivan Bassanini [1], Susanna Bertuletti [1], Sergio Riva [1], Chiara Tognoli [1,2], Marta Vanoni [1] and Daniela Monti [1,*]

[1] Istituto di Scienze e Tecnologie Chimiche "Giulio Natta", Consiglio Nazionale delle Ricerche, Via Mario Bianco 9, Milano, 20131, Italy

[2] Pharmaceutical Sciences Department, University of Milan, Via Mangiagalli 25, Milano, 20133, Italy

Table S1: Sequence analysis results of BLASTP alignments of Is2-SDR with a set of HSDHs.

Description	Scientific Name	Max Score	Total Score	Query Cover	E value	Per. Ident	Acc. Len	Accession
Bsp7alpha-HSDH 7-alpha-hydroxysteroid dehydrogenase [Bosea sp. 12-68-7] GenBank: OYW60865.1		145	145	96%	2e-45	38.49%	255	Query_793
11Psp3alpha-HSDH gi|835501872|ref|WP_047529067.1| 3-alpha-hydroxysteroid dehydrogenase [Pseudomonas sp. 11/12A]		76.6	76.6	39%	1e-19	38.24%	255	Query_775
Gri7alpha-HSDH 7-alpha-hydroxysteroid dehydrogenase [Grimontia sp. AD028] WP_046304034.1		139	139	96%	2e-43	36.84%	255	Query_795
Bs7alpha-HSDH 7-alpha-hydroxysteroid dehydrogenase [Brevundimonas] WP_046653274.1		134	134	96%	2e-41	36.76%	253	Query_794
Pa7alpha-HSDH 7-alpha-hydroxysteroid dehydrogenase [Pseudoruegeria aquimaris] WP_085868047.1		139	139	96%	3e-43	36.07%	255	Query_797
Csc7alpha-HSDH AAB61151.1 7-alpha-hydroxysteroid dehydrogenase [Clostridium scindens]		143	143	96%	1e-44	36.03%	266	Query_788
Rg3alpha-HSDH UniProtKB/Swiss-Prot: A7B3K3.1 bile acid 7-dehydroxylase 1/3 [[Ruminococcus] gnavus CAG:126] GenBank…		133	133	96%	3e-41	36.00%	250	Query_772
Esp3alpha-HSDH CDD59474.1 7-alpha-hydroxysteroid dehydrogenase [Eggerthella sp. CAG:298]		132	132	96%	2e-40	35.89%	250	Query_770
Ls12alpha-HSDH SDR family oxidoreductase [Lysinibacillus sphaericus] WP_036222017.1		99.8	99.8	96%	4e-28	35.86%	251	Query_764
Ka7alpha-HSDH 7-alpha-hydroxysteroid dehydrogenase [Kaistia adipata] WP_029074103.1		136	136	96%	3e-42	35.46%	255	Query_796
2Sm7alpha-HSDH 7-alpha-hydroxysteroid dehydrogenase [Shewanella] WP_076500293.1		132	132	96%	2e-40	35.39%	255	Query_792
El3beta-HSDH UniProtKB/Swiss-Prot: C8WJW0.1 NAD-dependent bile acid 3-beta-dehydrogenase [Eggerthella lenta]		130	130	97%	6e-40	35.22%	261	Query_777
Cso7alpha-HSDH AAA53556.1 7-alpha-hydroxysteroid dehydrogenase [Clostridium sordellii]		145	145	97%	2e-45	34.94%	267	Query_787
El12alpha-HSDH WP_114518444.1 SDR family oxidoreductase [Eggerthella lenta]		108	108	96%	1e-31	34.82%	255	Query_765
Cd7alpha-HSDH AVB35871.1 SDR family NAD(P)-dependent oxidoreductase [Clostridioides difficile]		138	138	96%	8e-43	34.80%	262	Query_790
Ca7alpha-HSDH AET80685.1 7-alpha-hydroxysteroid deydrogenase [Clostridium sardiniense]		137	137	98%	1e-42	34.40%	262	Query_789
Chy12alpha-HSDH WP_006441568.1 SDR family oxidoreductase [[Clostridium] hylemonae]		129	129	96%	2e-39	34.39%	259	Query_767
Ec7alpha-HSDH KXH01569.1 7-alpha-hydroxysteroid dehydrogenase [Escherichia coli]		132	132	96%	1e-40	34.16%	255	Query_785
Csp12alpha-HSDH Sequence 12 from patent US 9096825, GenBank: AOB10242.1,WP_044992937.1		127	127	96%	1e-38	33.60%	270	Query_762
Ea7alpha-HSDH 7-alpha-hydroxysteroid dehydrogenase [Erythrobacter atlanticus] WP_048884278.1		121	121	96%	2e-36	33.33%	255	Query_791
Rg3beta-HSDH UniProtKB/Swiss-Prot: A7AZH2.1 3-beta-hydroxycholanate 3-dehydrogenase (NADP(+)) [Ruminococcus gnav…		134	134	97%	3e-41	33.21%	276	Query_779
Bf7alpha-HSDH SDR family oxidoreductase [Bacteroides fragilis] WP_032598793.1		135	135	97%	6e-42	33.20%	259	Query_786
2El3beta-HSDH UniProtKB/Swiss-Prot: C8WGQ3.1 NAD-dependent bile acid 3-beta-dehydrogenase [Eggerthella lenta]		125	125	98%	5e-38	33.07%	260	Query_778
Psp3alpha-HSDH gi|114793713|pdb|2DKN|A Chain A, Crystal Structure Of The 3-alpha-hydroxysteroid Dehydrogenase From …		68.6	68.6	45%	1e-16	33.04%	255	Query_774
Rr12alpha-HSDH WP_026137560.1 SDR family oxidoreductase [Rhodococcus ruber]		112	112	96%	5e-33	32.93%	250	Query_766
Chi12alpha-HSDH WP_006439999.1 MULTISPECIES: SDR family oxidoreductase [Clostridium] hiranonis DSM 13275		112	112	96%	9e-33	32.82%	266	Query_768
Ct3alpha-HSDH gi|815828503|ref|WP_046461354.1| 3-alpha-hydroxysteroid dehydrogenase [Comamonas testosteroni]		64.3	64.3	49%	3e-15	32.80%	257	Query_773
Sm7alpha-HSDH KRG42928.1 7-alpha-hydroxysteroid dehydrogenase [Stenotrophomonas maltophilia]		94.4	94.4	96%	4e-26	32.11%	258	Query_784
Hh7alpha-HSDH 7-alpha-hydroxysteroid dehydrogenase [Halomonas halodenitrificans] WP_027961750.1		119	119	96%	1e-35	32.10%	255	Query_783
Cs12alpha-HSDH EDS06338.1 oxidoreductase, short chain dehydrogenase/reductase family protein [[Clostridium] scindens A…		105	105	96%	2e-30	31.68%	266	Query_769
Esp12alpha-HSDH CDD59475.1 putative uncharacterized protein [Eggerthella sp. CAG:298]		105	105	96%	3e-30	31.54%	266	Query_763
El3alpha-HSDH UniProtKB/Swiss-Prot: C8WMP0.1 SDR family oxidoreductase [Eggerthella] WP_009306474.1		52.4	52.4	38%	5e-11	31.07%	259	Query_771
Dm7alpha-HSDH glucose 1-dehydrogenase [Deinococcus marmoris] WP_075832535.1		99.8	99.8	96%	4e-28	30.71%	260	Query_782
Ct3beta-HSDH gi|309860|gb|AAA25742.1| beta-hydroxysteroid dehydrogenase [Comamonas testosteroni]		97.8	97.8	95%	2e-27	30.08%	254	Query_780
ngi1_7alpha-HSDH		100	100	96%	2e-28	29.69%	263	Query_781
Hh7beta -HSDH SDR family oxidoreductase [Halomonas halodenitrificans] WP_027961749.1		72.8	72.8	96%	4e-18	26.10%	266	Query_751
Bsp7beta-HSDH MULTISPECIES: SDR family oxidoreductase [Brucella] WP_004684107.1		74.7	74.7	96%	7e-19	25.91%	264	Query_752
Rt7beta-HSDH Short-chain dehydrogenases of various substrate specificities [[Ruminococcus torques L2-14] GenBank: CBL2…		42.4	42.4	73%	1e-07	25.79%	264	Query_757
Lm7beta-HSDH SDR family NAD(P)-dependent oxidoreductase [Libanicoccus massiliensis] WP_073294202.1		55.8	55.8	74%	3e-12	25.77%	267	Query_759
Ca7beta-HSDH 7-beta-hydroxysteroid dehydrogenase [Clostridium sardiniense] GenBank: AET80684.1		49.7	49.7	73%	4e-10	25.13%	261	Query_761
Cn7beta-HSDH SDR family NAD(P)-dependent oxidoreductase [Clostridium nigeriense] WP_066892209.1		55.8	55.8	73%	3e-12	24.62%	263	Query_758
Rs7beta-HSDH SDR family oxidoreductase [Rhodobacter sphaeroides] WP_011911126.1		71.2	71.2	97%	1e-17	24.19%	264	Query_753
Cae7beta-HSDH tr|A4ECA9|A4ECA9_9ACTN 7bHSDH OS=Collinsella aerofaciens SDR family NAD(P)-dependent oxidoredu…		47.8	47.8	73%	2e-09	23.96%	263	Query_755
Sc7beta-HSDH SDR family oxidoreductase [Stanieria cyanosphaera] WP_015212061.1 rf 1 NC_019765.1:c215210-214413 St…		66.6	66.6	96%	6e-16	23.72%	265	Query_750
Ls7beta-HSDH WP_045806907.1 SDR family NAD(P)-dependent oxidoreductase [Lactobacillus spicheri]		37.0	37.0	73%	7e-06	22.96%	264	Query_754
Rg7beta-HSDH SDR family NAD(P)-dependent oxidoreductase [[Ruminococcus gnavus] WP_004843516.1		37.4	37.4	73%	4e-06	22.00%	263	Query_756
El7beta-HSDH SDR family NAD(P)-dependent oxidoreductase [Eubacterium limosum] WP_038354045.1		38.5	38.5	73%	2e-06	21.35%	265	Query_760

Scheme S1: Spectrophotometric assay of ethyl 3-methyl-2-oxobutyrate (EMOB) reduction used for Is2-SDR functional characterization.

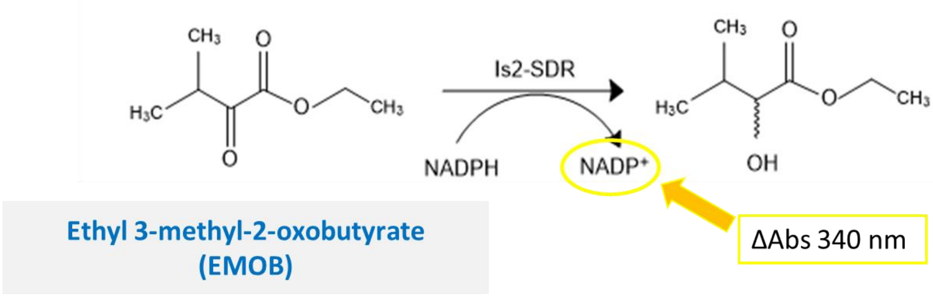

Figure S1: Is2-SDR activity at different pH values.

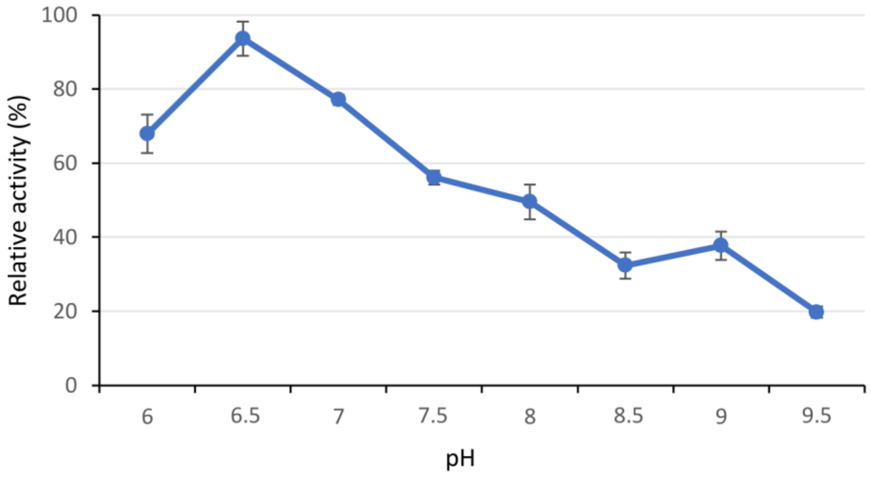

Figure S2: Is2-SDR activity at different temperatures.

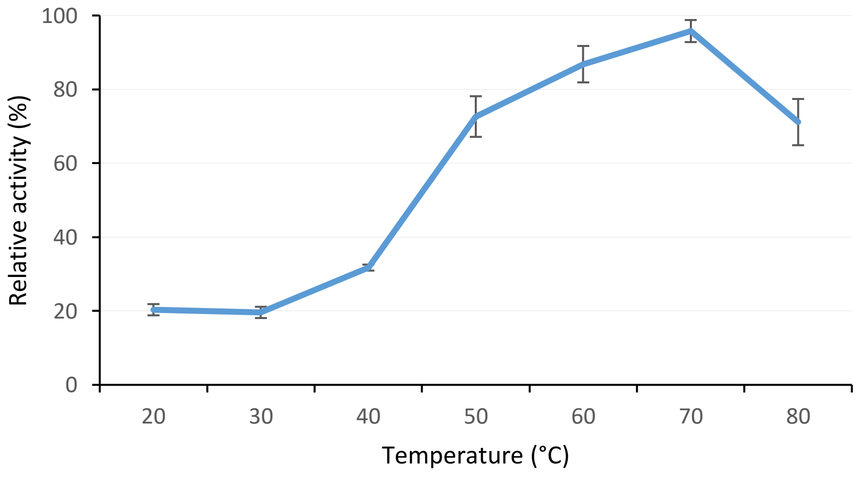

Table S2: Chemical structure of substrates/products from Table 1.

	Substrate	Products	Enantiomeric excess (e.e., %)
**17**	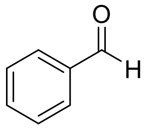	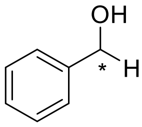	--
**18**	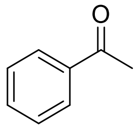	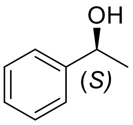 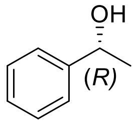	91.0 (*S*)
**19**	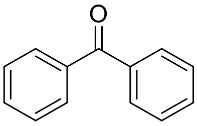	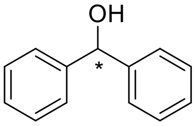	--
**22**	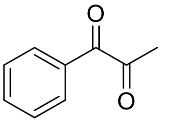	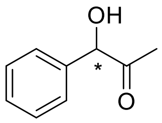 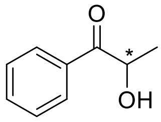 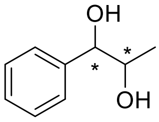	n.d.
**23**	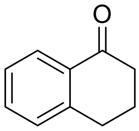	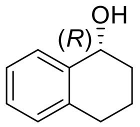 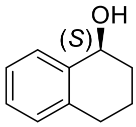	98 *(R)*
**24**	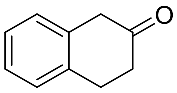	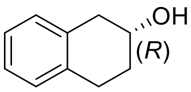 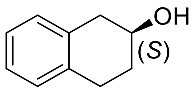	22 *(R)*
**29**	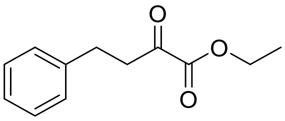	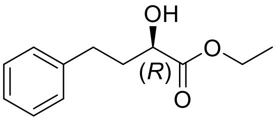	> 99 *(R)*

Figure S3: Chemical structure of the bile acids tested during Is2-SDR characterization. No oxidative activity was observed in the presence of compounds A-F, thus suggesting the lack of either 3α-, 3β-, 7α-, 7β-, or 12α-HSDH activity. Reductive activity was detected only in the presence of derivatives showing a keto group in position 3 (compounds G-I, last row).

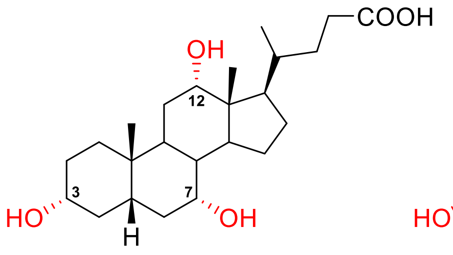

A) Cholic acid

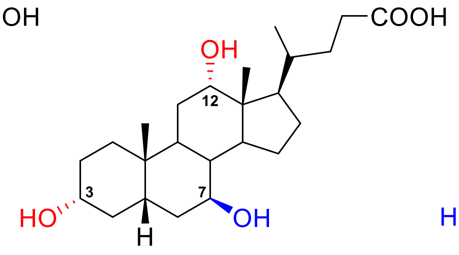

B) Ursocholic acid

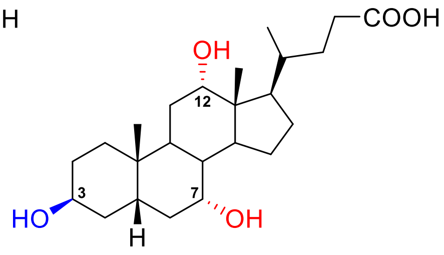

C) Isocholic acid

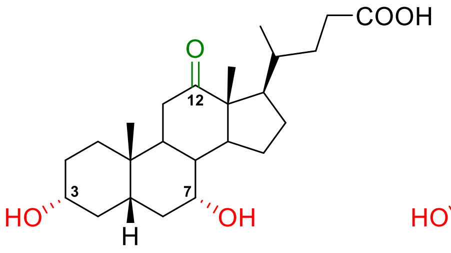

D) 12-Ketochenodeoxycholic acid

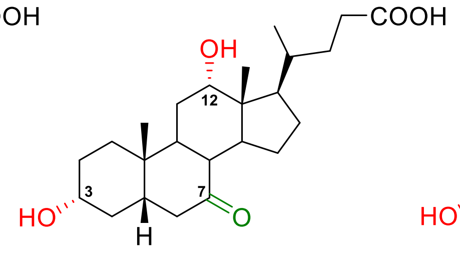

E) 3α,12α-Dihydroxy-7-oxo-5β-cholanoic acid

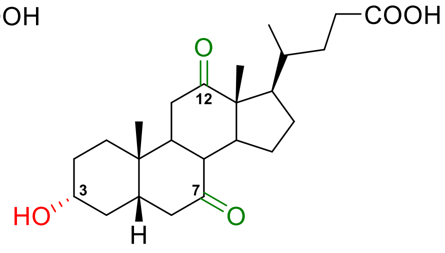

F) 3α-Hydroxy-7,12-dioxo-5β-cholanoic acid

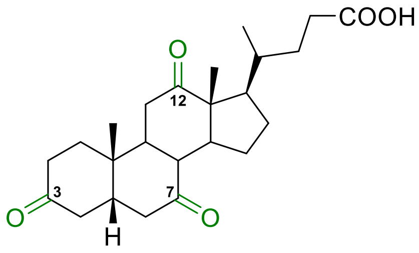

G) Dehydrocholic acid

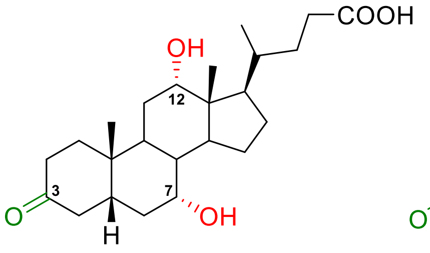

H) 7α,12α-Dihydroxy-3-oxo-5β-cholanoic acid

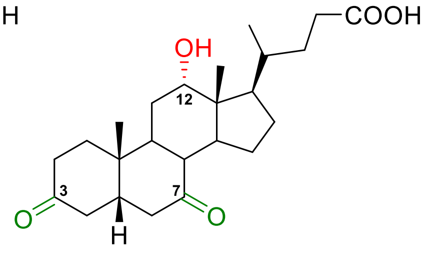

I) 12α-Hydroxy-3,7-dioxo-5β-cholanoic acid

Figure S4: Reduction of dehydrocholic acid (**30**) catalyzed by Is2-SDR: NMR Spectra.

a) ^{1}H NMR

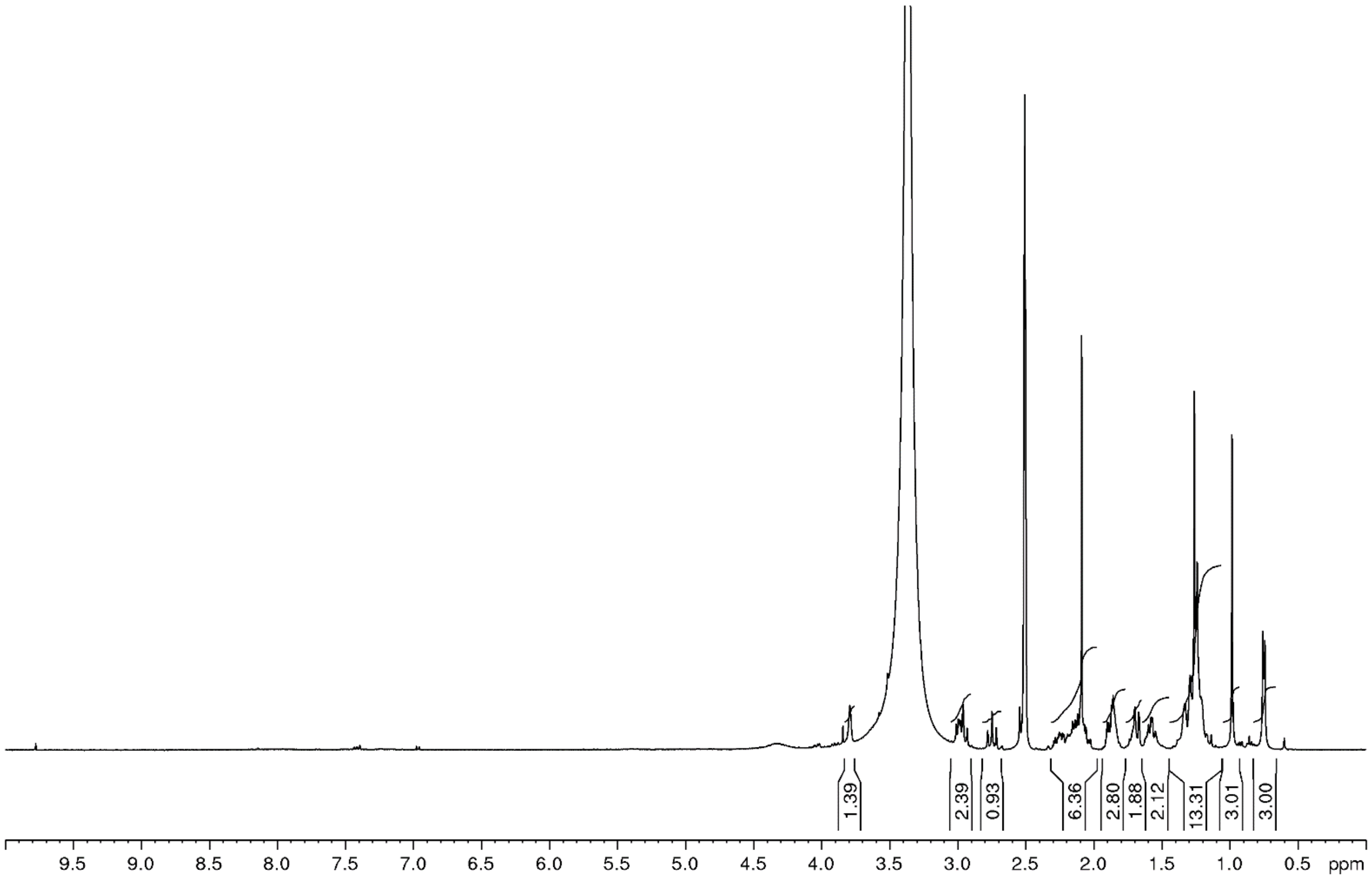

b) ^{1}H NMR expansion

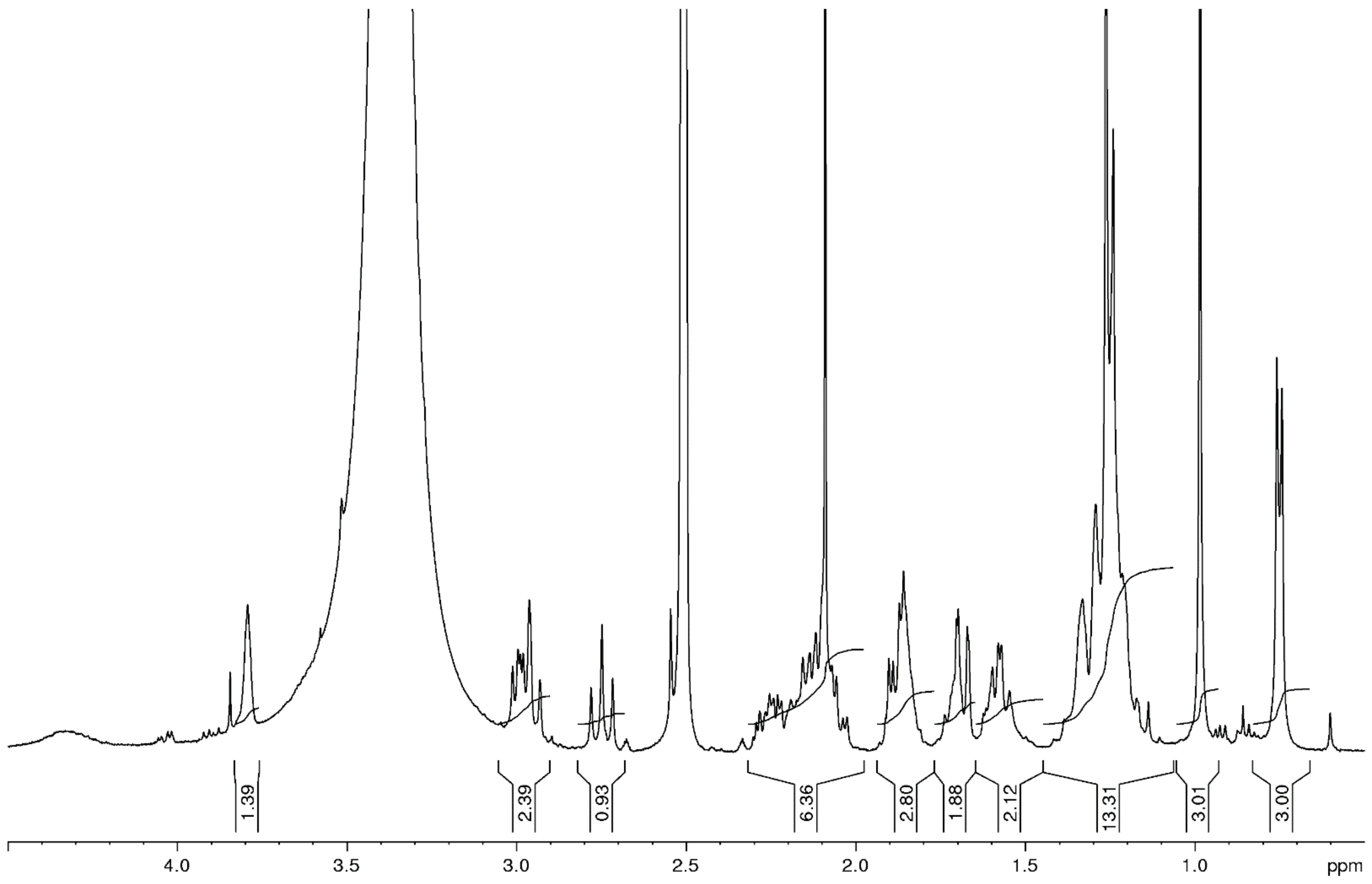

c) ^{13}C NMR APT

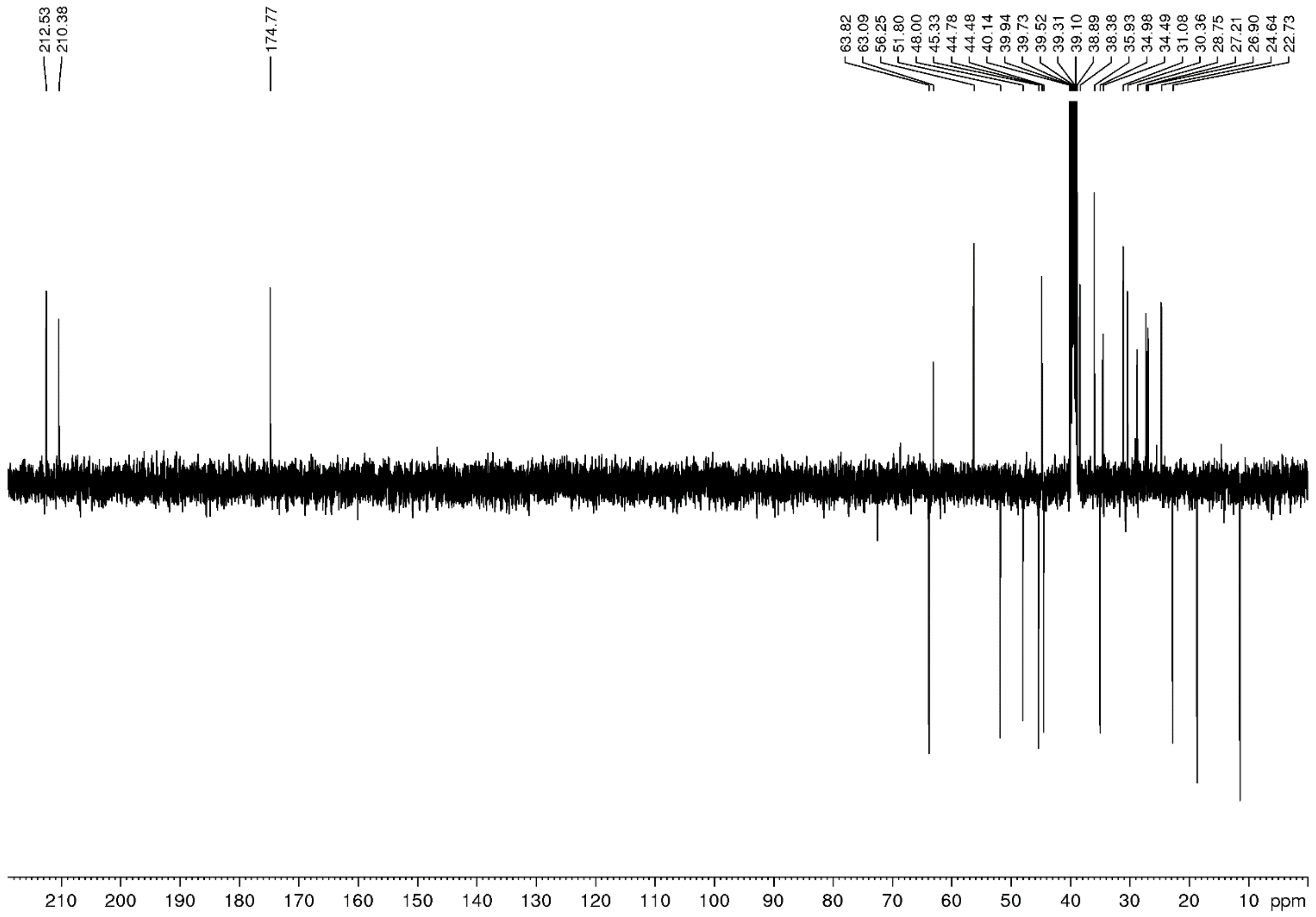
212.53
210.38
174.77
63.82
63.09
56.25
51.80
48.00
45.33
44.78
44.48
40.14
39.94
39.73
39.52
39.31
39.10
38.89
38.38
35.93
34.98
34.49
31.08
30.36
28.75
27.21
26.90
24.64
22.73
210
200
190
180
170
160
150
140
130
120
110
100
90
80
70
60
50
40
30
20
10
ppm

d) ^{13}C NMR APT expansion

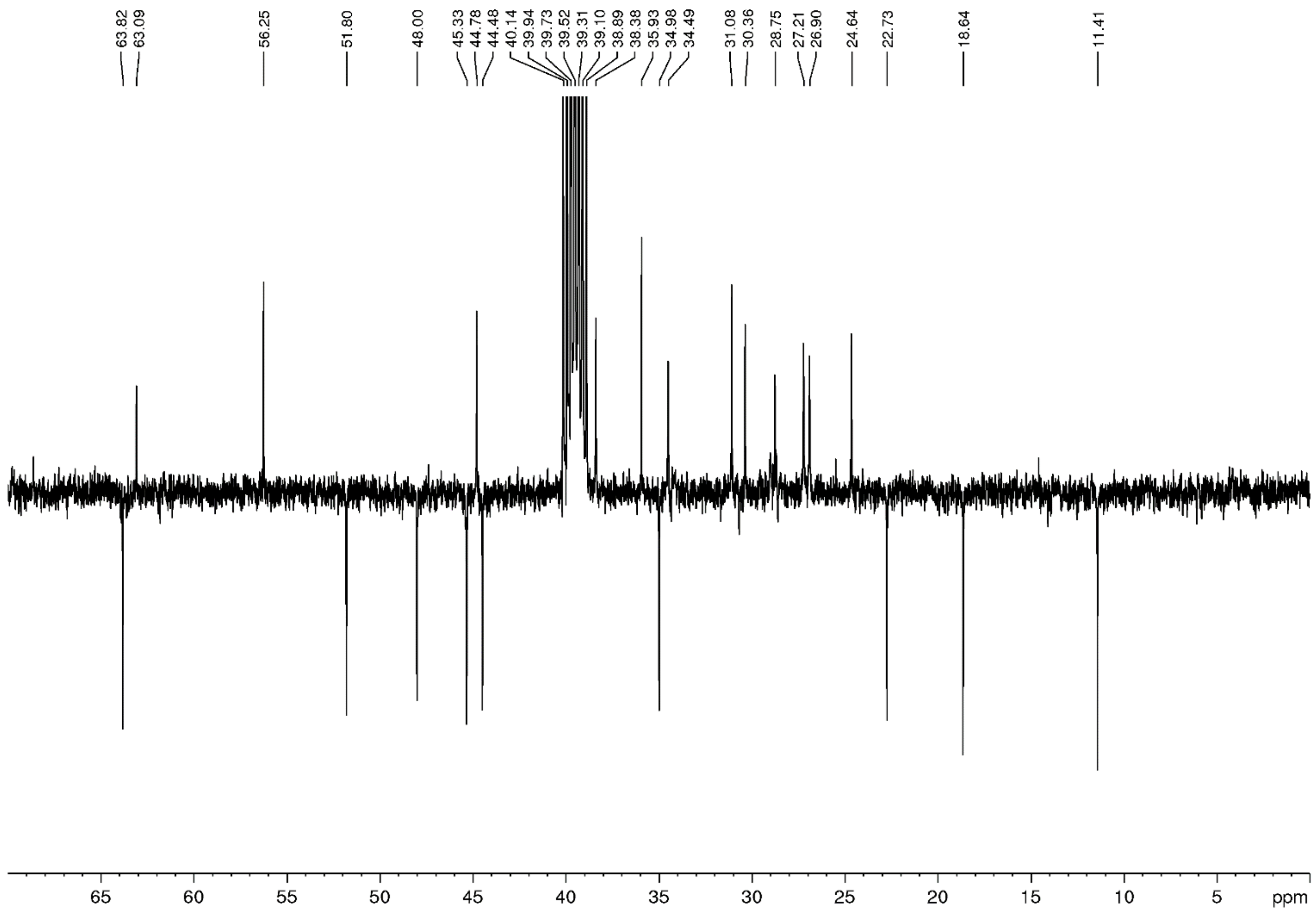

e) ^{13}C NMR DEPT

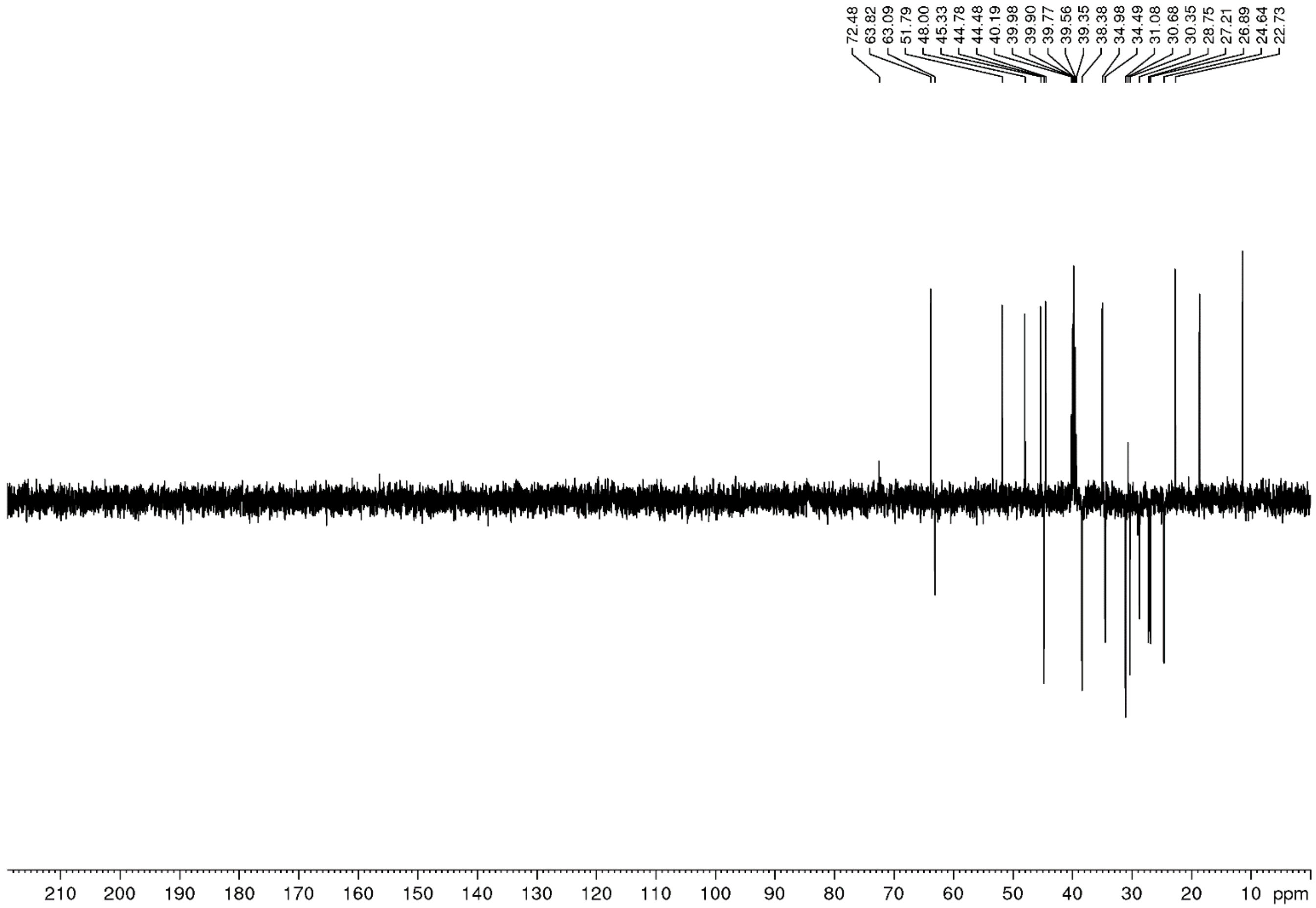

f) HSQC

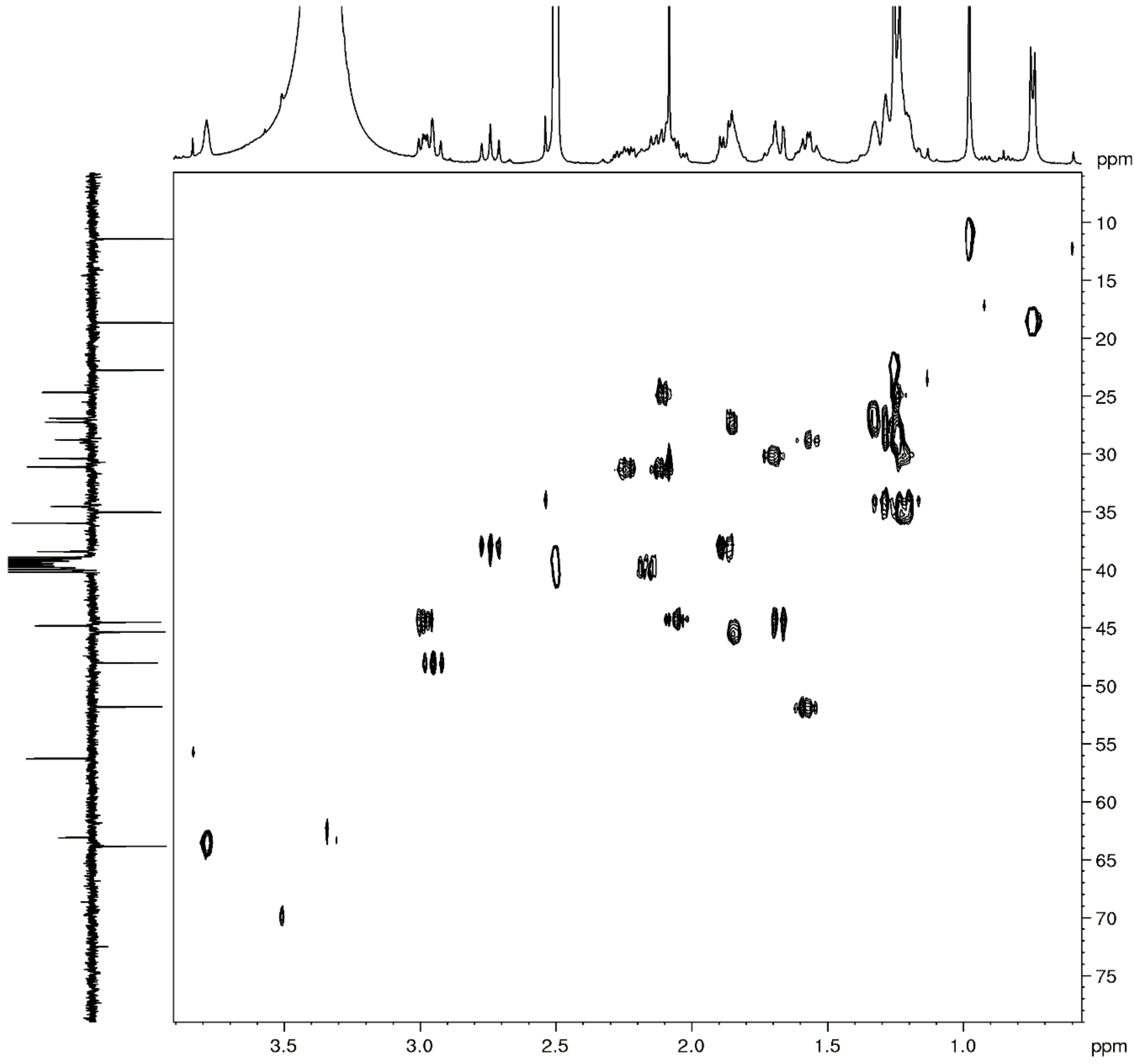

g) HMBC

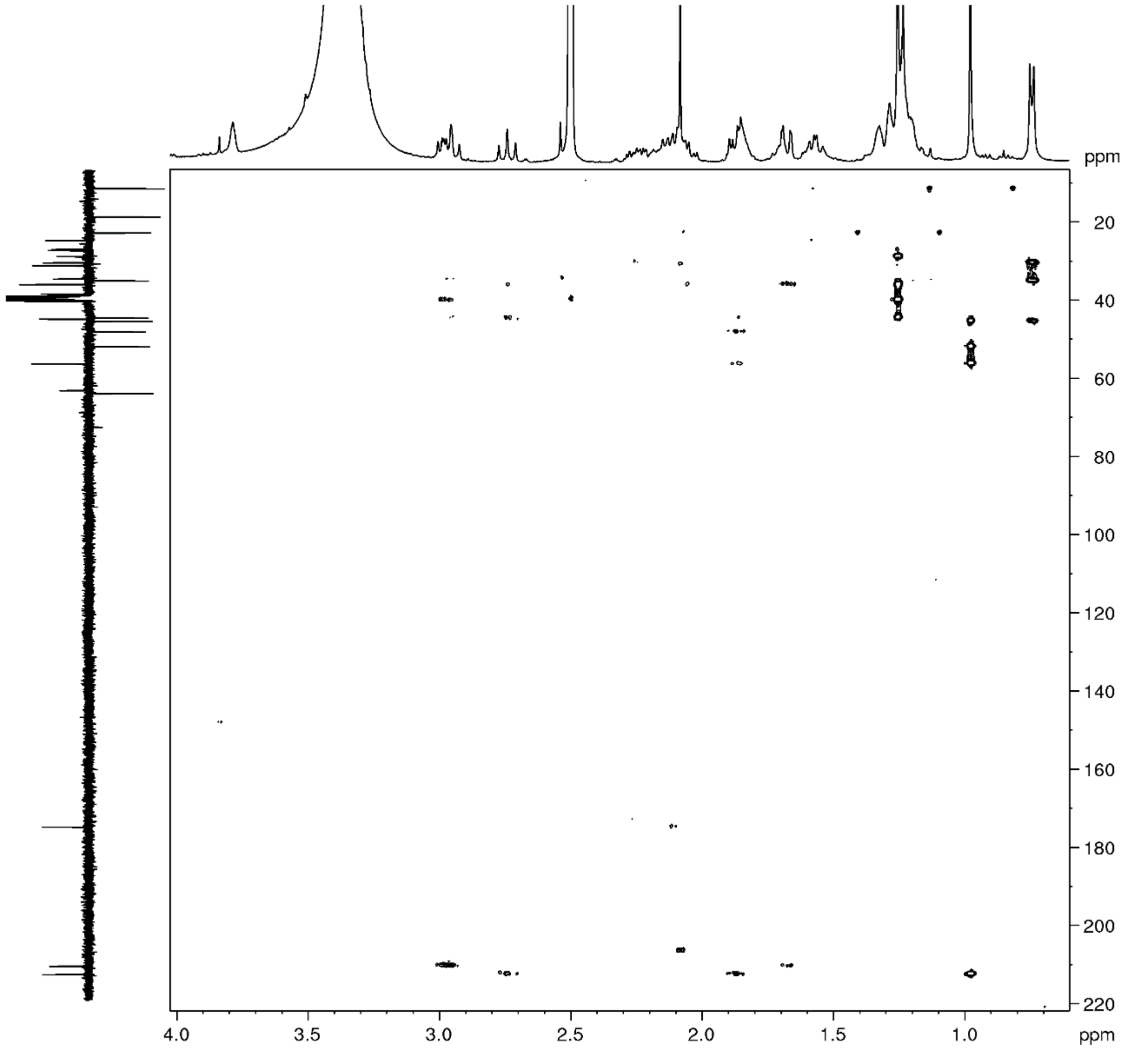

Figure S5: Is2-SDR-catalyzed reduction of 5α-dihydrotestosterone (**31**): NMR spectrum, TLC analysis

a) ^{1}H NMR

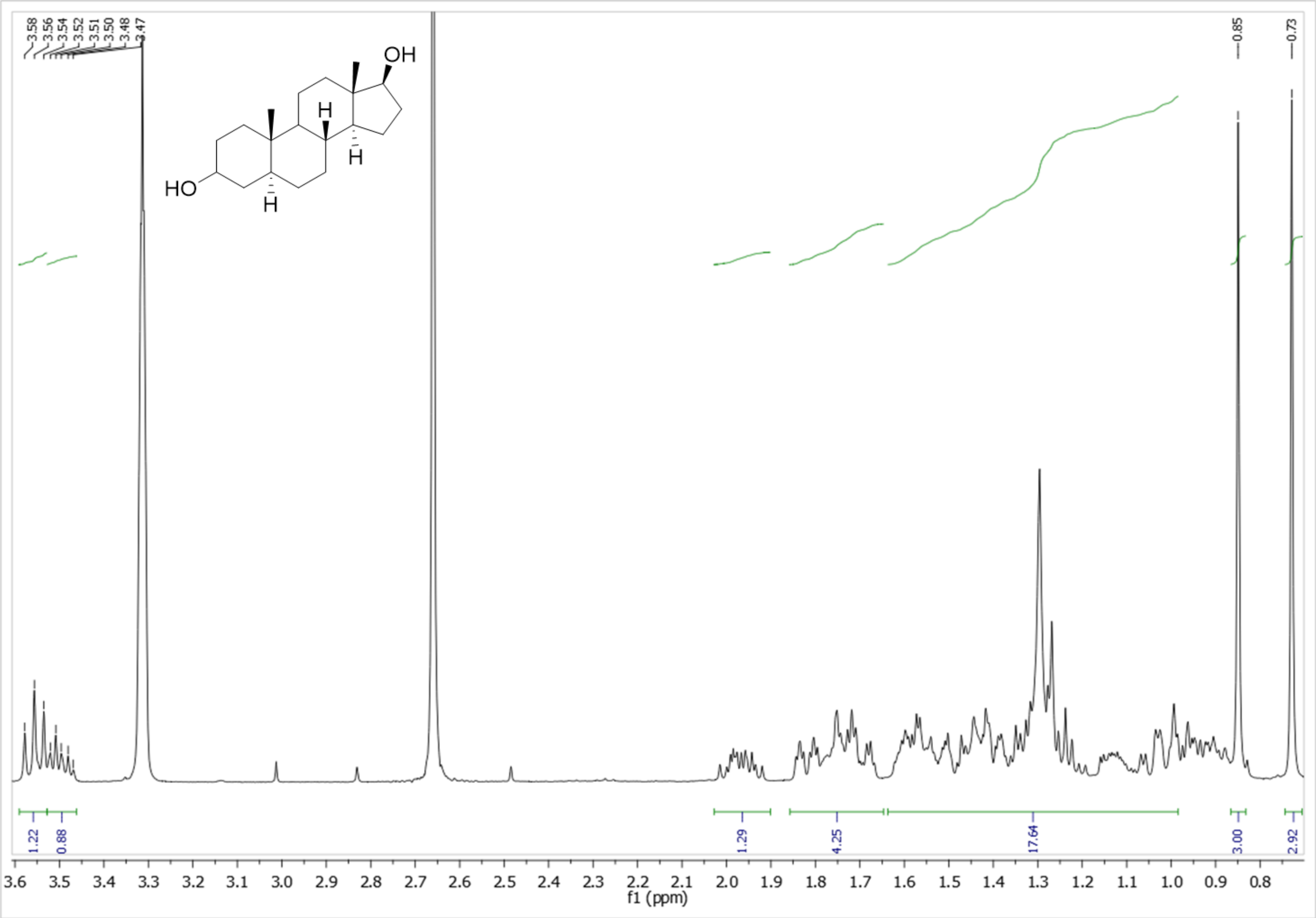

b) TLC analysis

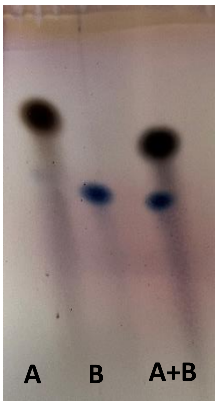

A: 5α-dihydrotestosterone

B: 5α-androstane-3β,17β-diol

Figure S6: Is2-SDR-catalyzed reduction of androsterone (**32**): NMR spectrum, TLC analyses

a) ^{1}H NMR

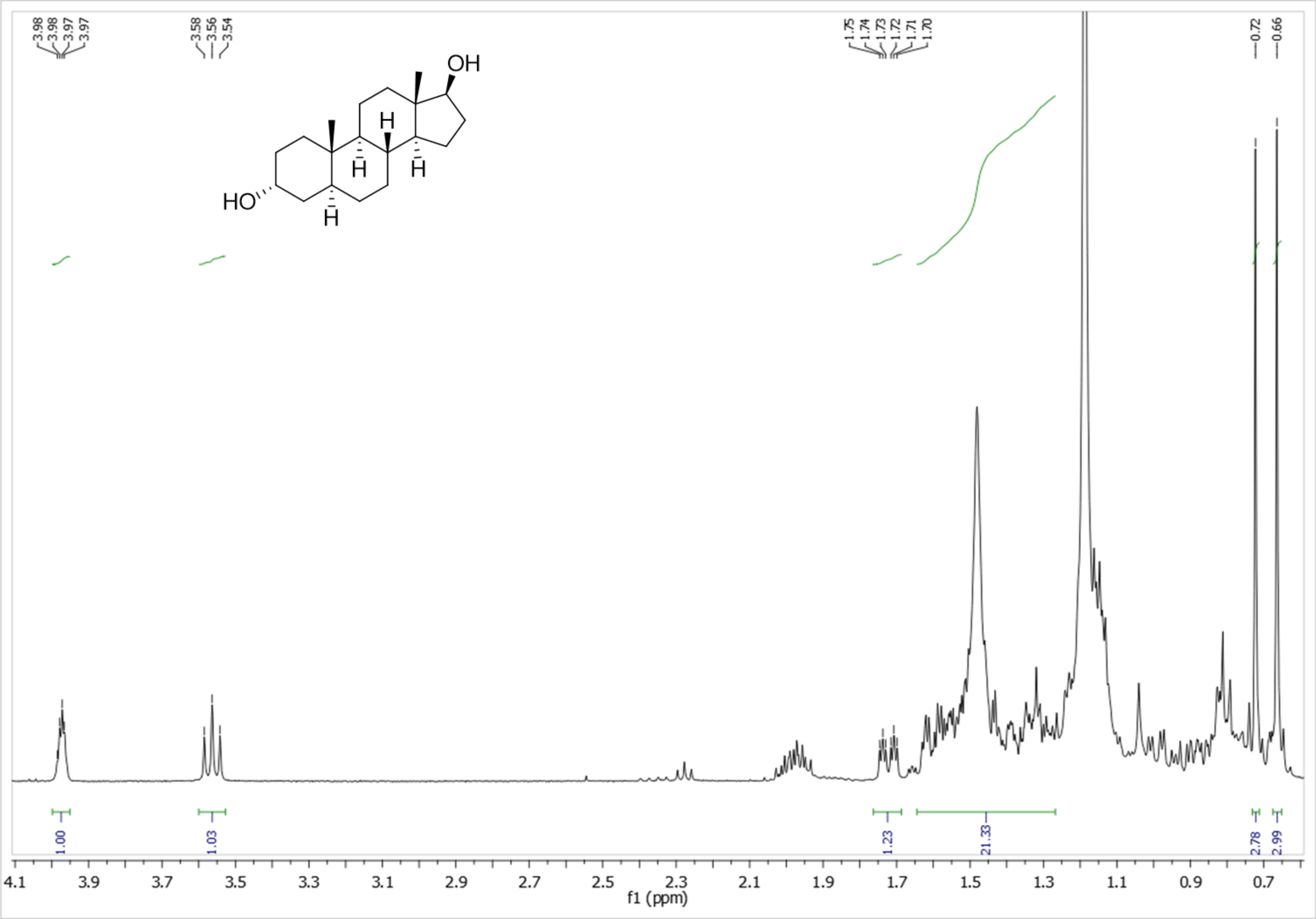

b) TLC analysis

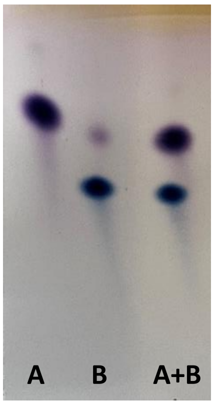

A: androsterone

B: 5α-androstane-3α,17β-diol

Figure S7: Sequence alignment of Is2-SDR with Lm-FabG. Blue stars indicate the conserved residues in cofactor binding, while red stars show the amino acids of the catalytic triad.

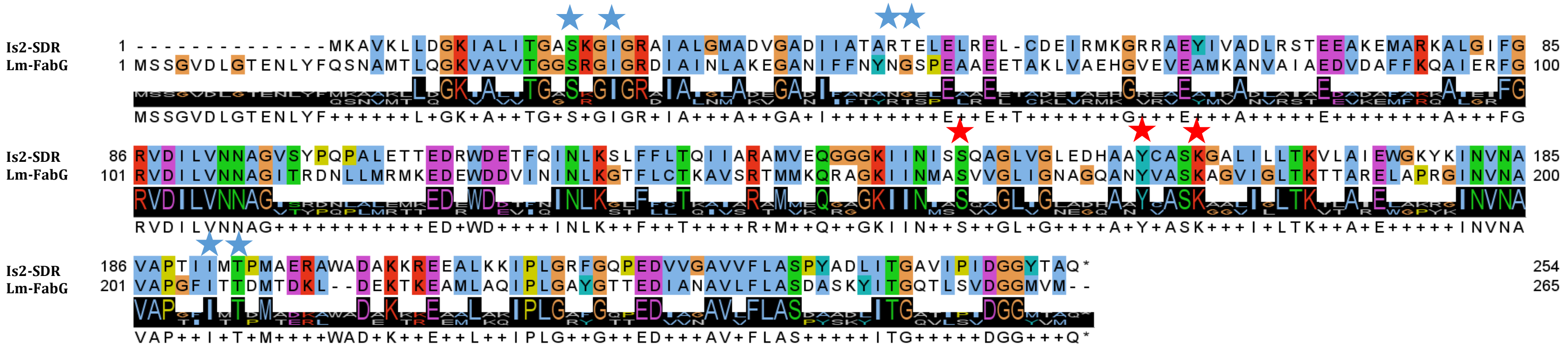

Figure S8: Is2-SDR-catalyzed reduction of benzaldehyde (**17**): GC spectra

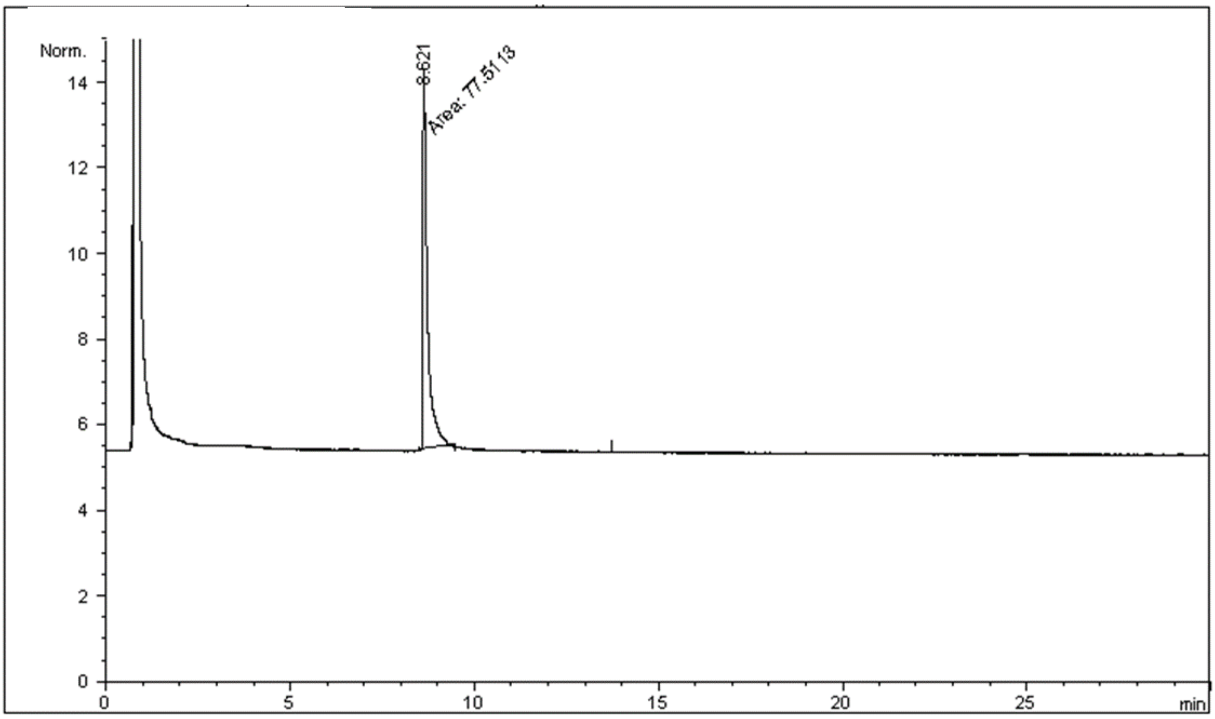

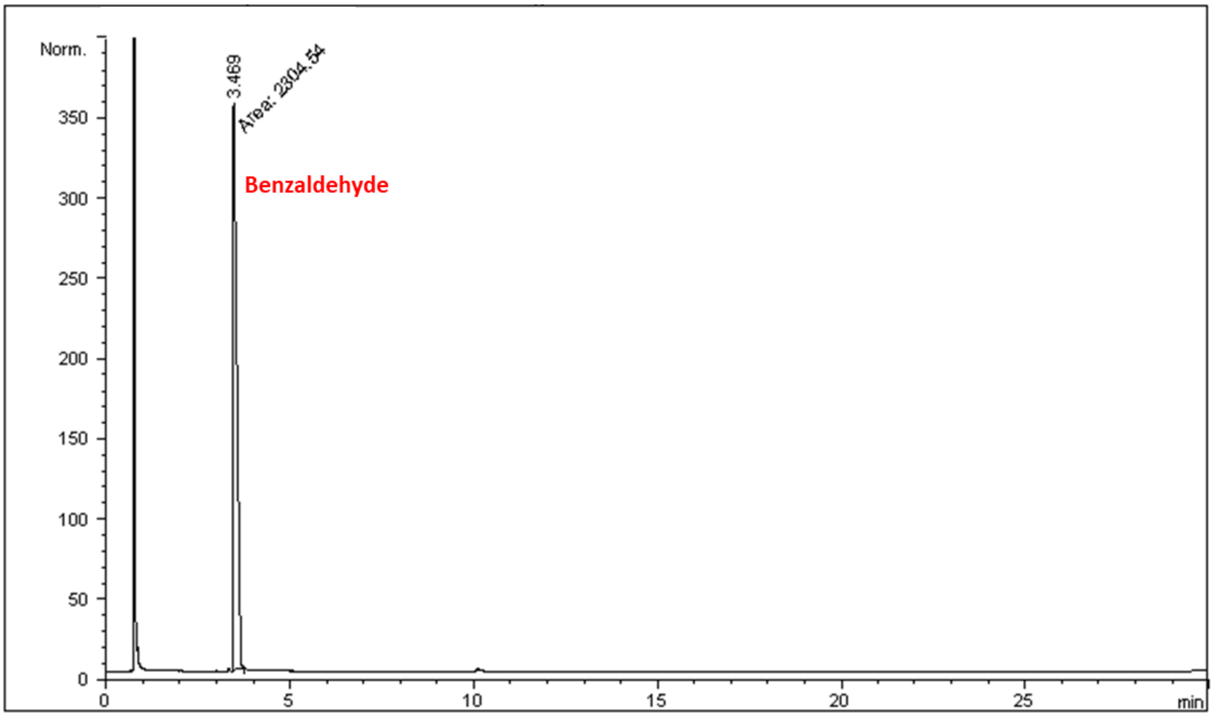

Figure S9: Is2-SDR-catalyzed reduction of acetophenone (**18**): GC spectrum

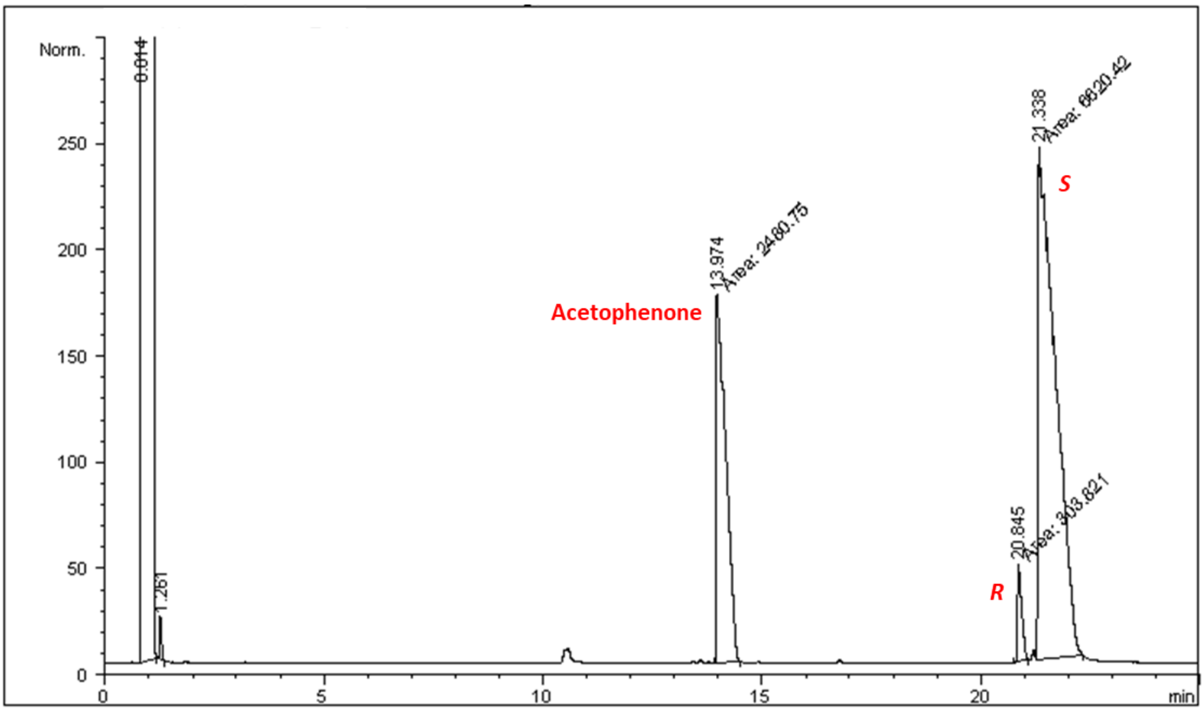

Figure S10: Is2-SDR-catalyzed reduction of ketone benzophenone (**19**): GC-MS spectrum

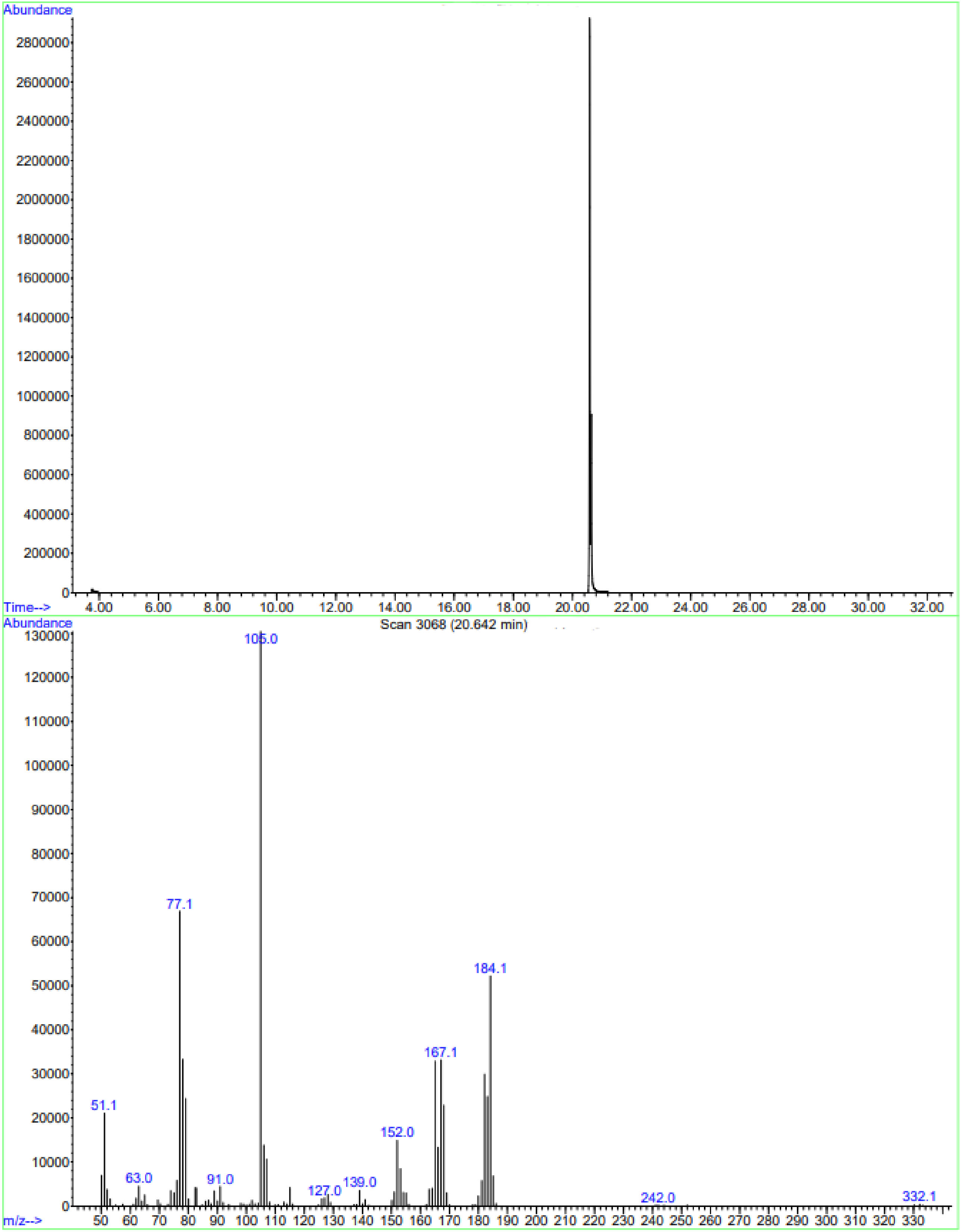

Figure S11: Is2-SDR-catalyzed reduction of 1,2-diketone 1-phenylpropane-1,2-dione (**22**): NMR spectra.

a) ^{1}H NMR

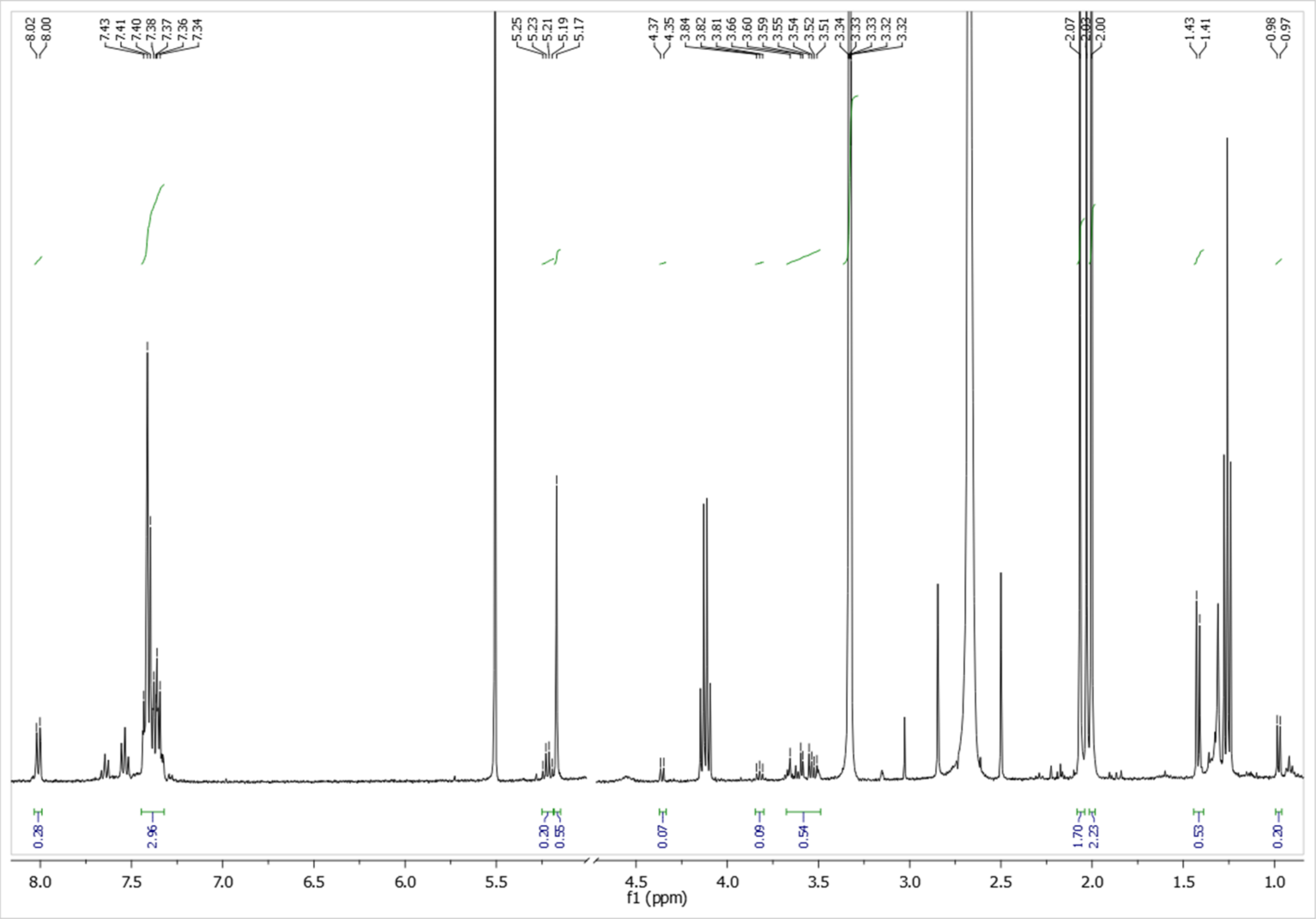

b) ^{1}H NMR expansion

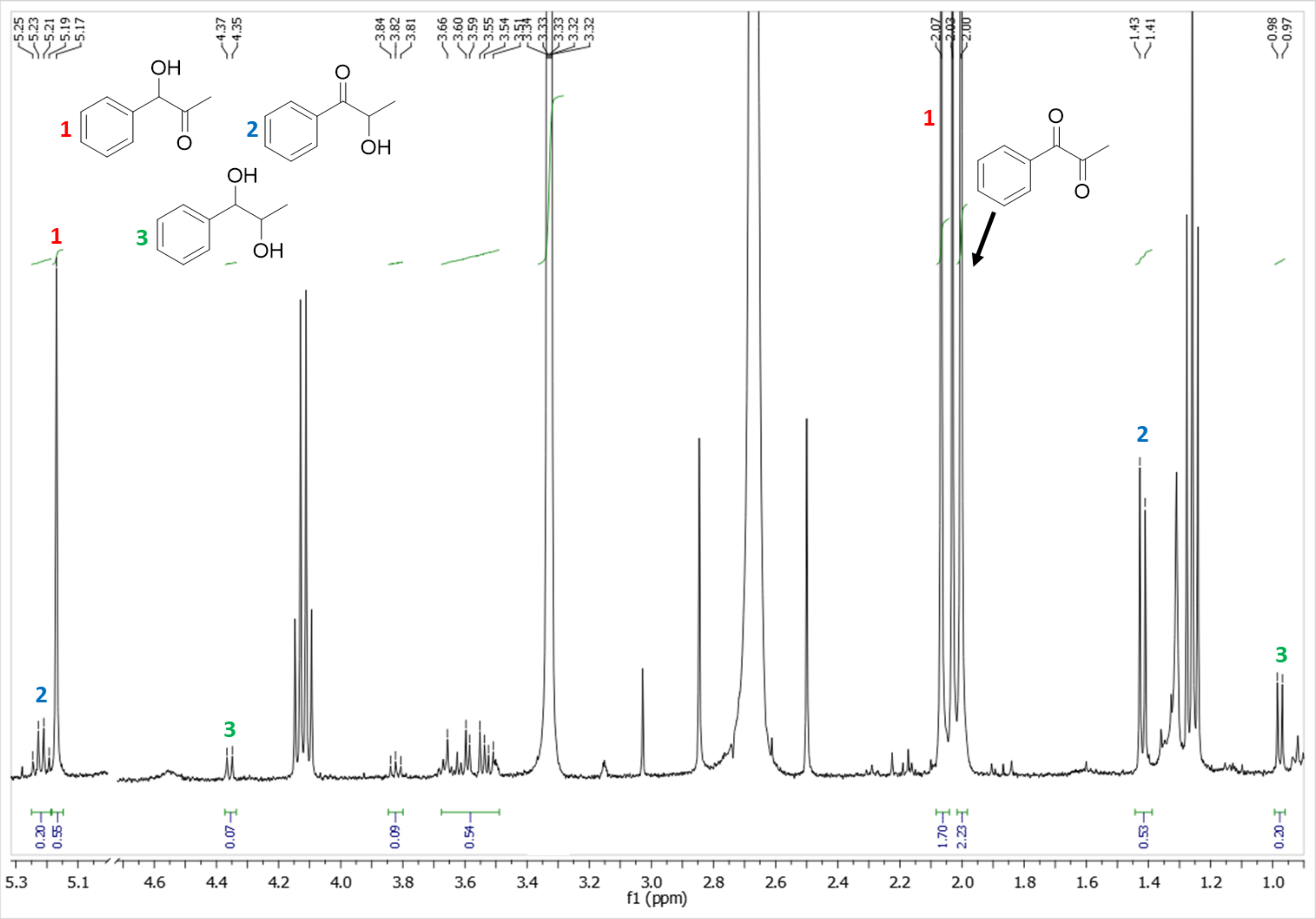

Figure S12: Is2-SDR-catalyzed reduction of α-tetralone (**23**): GC spectrum

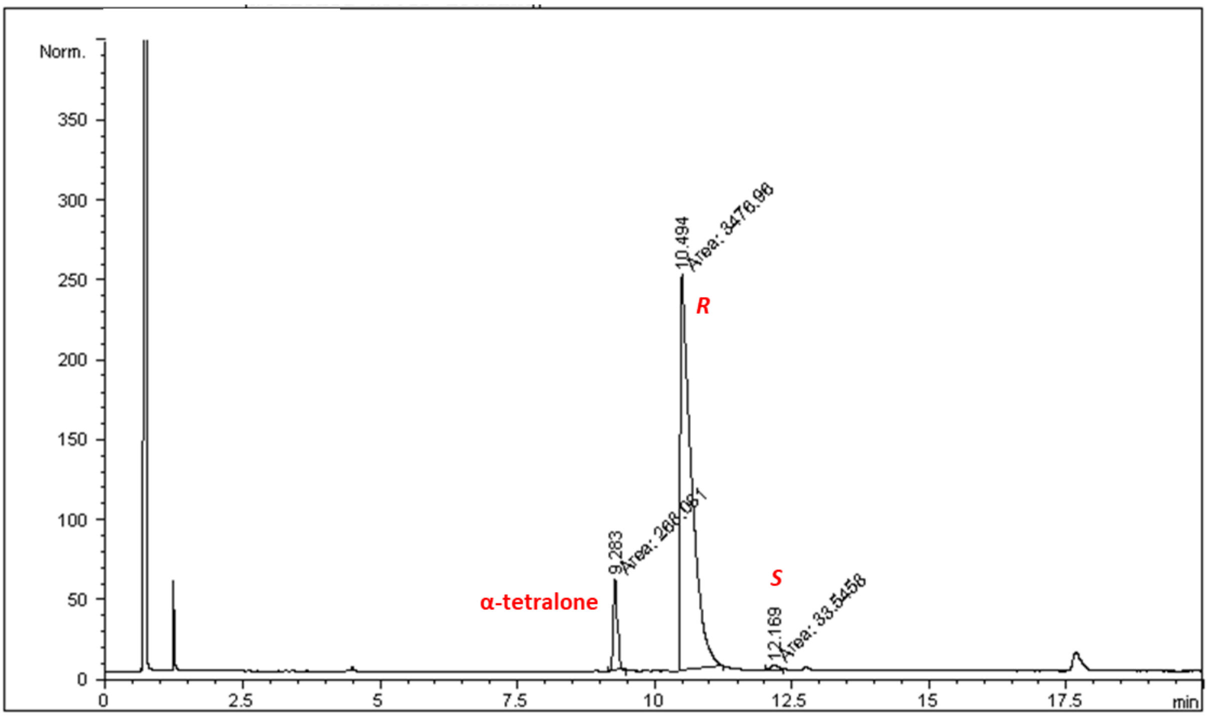

Figure S13: Is2-SDR-catalyzed reduction of β-tetralone (**24**): GC spectrum

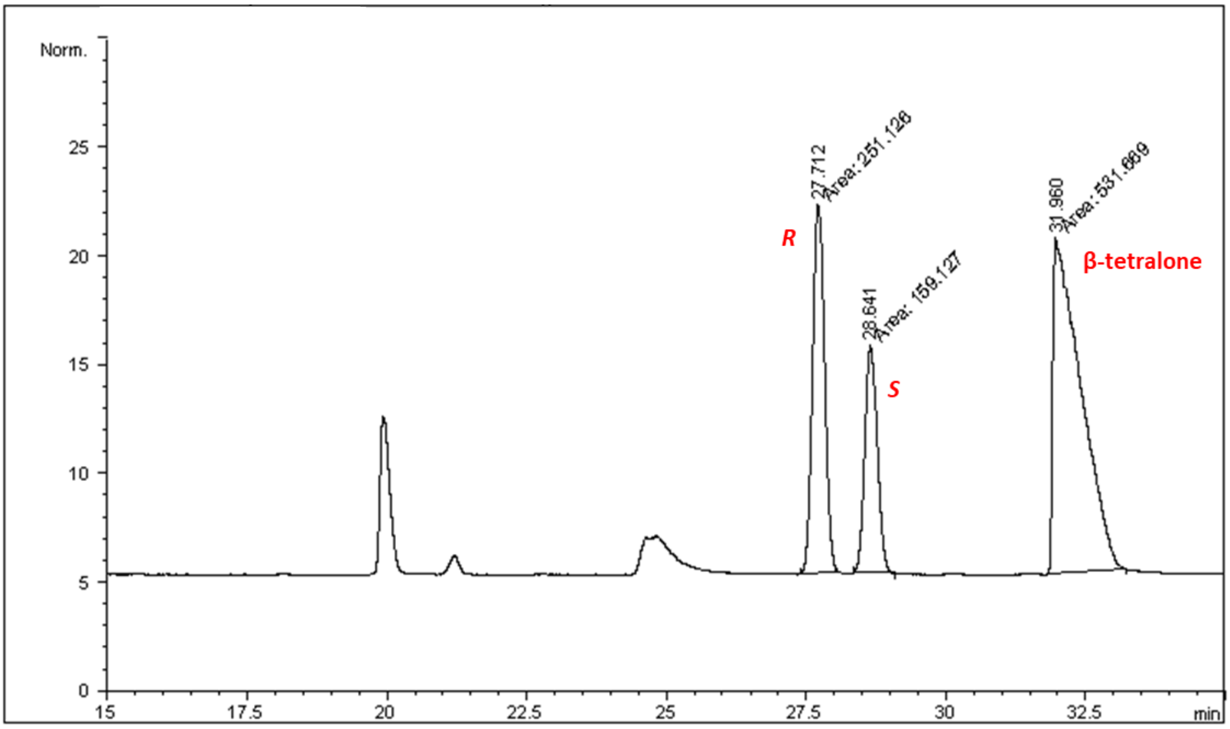

Figure S14: Is2-SDR-catalyzed reduction of ethyl 2-oxo-4-phenylbutanoate (**29**): HPLC spectrum

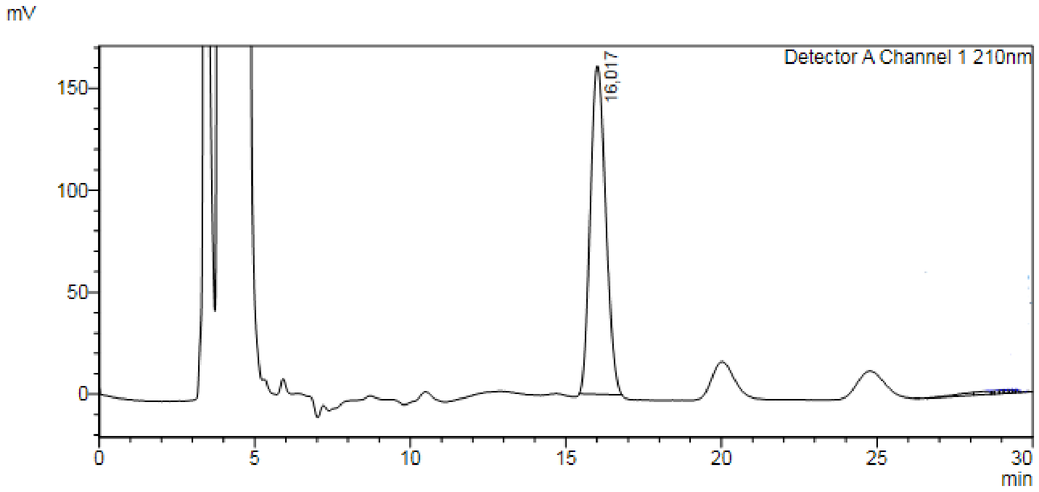

Supplement: Supplementary file 1 [file ijms-23-12153-s001.zip › ijms-1947194-supplementary.pdf]
